# Supplementary material for: The plant pathogenic bacterium Candidatus Liberibacter solanacearum induces calcium-regulated autophagy in midgut cells of its insect vector Bactericera trigonica
Source: Microbiol Spectr. 2023 Sep 28;11(5):e01301-23. doi: 10.1128/spectrum.01301-23 (PMC10581152; doi:10.1128/spectrum.01301-23)
Supplement: Supplemental figures — Fig. S1 to S5. [file spectrum.01301-23-s0001.pdf]

# Supplementary figures

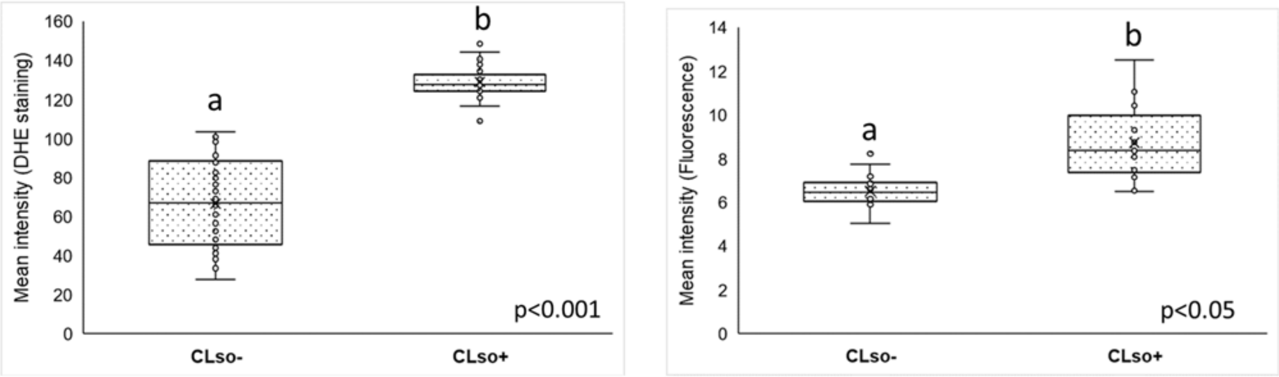


**Figure S1**. Intensity measurements for ROS for the light microscopy and the confocal images using ImageJ. (A) Mean intensity measurements for DHE-staining of midguts under light microscope. (B) Mean intensity measurement for DHE-staining under confocal microscope. The significance between samples are denoted by alphabets.

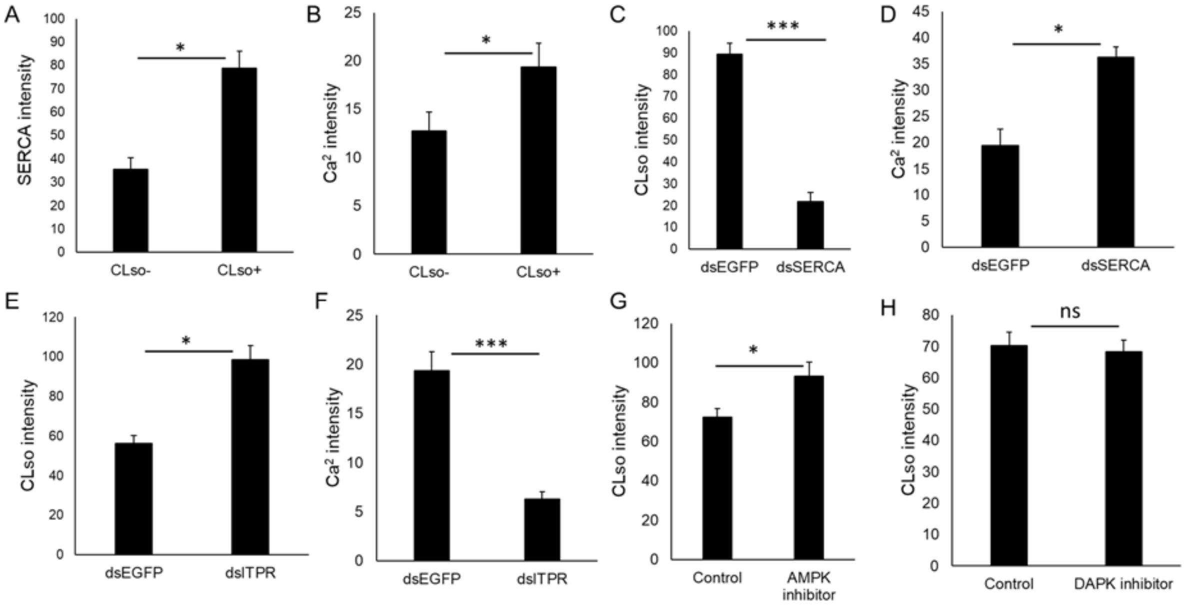


**Figure S2.** Intensity measurements for the confocal images using ImageJ. A. Immunostaining signals for SERCA antibody. B. Calcium signals for Fluo-8AM. C. Immunostaining signals for CLso following dsSERCA treatment. D. Calcium signals following dsSERCA treatment. E. Immunostaining signls for CLso after dsITPR treatment. F. Calcium signals following dsITPR treatment. G. Immunostaining signals for CLso following AMPK inhibitor. H. Immunostaining signals for CLso after DAPK inhibitor. * denotes $p \leq 0.05$, *** denotes $p \leq 0.01$; Error bars denote SE with $n \geq 7$.

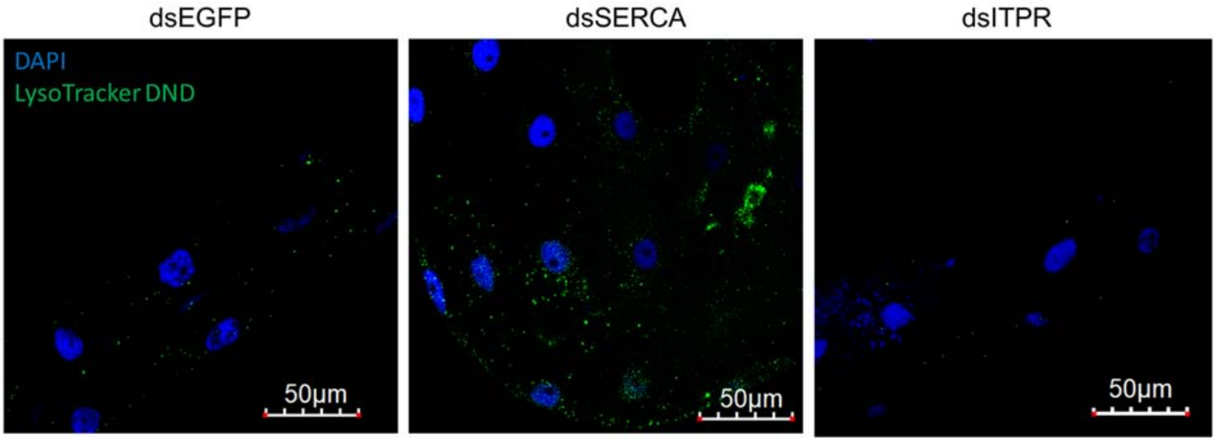


**Figure S3.** Detection of autolysosomes with Lysotracker DND (green) in dsSERCA and dsITPR treated midguts in comparision with dsEGFP treated midguts, counterstained with DAPI (blue).

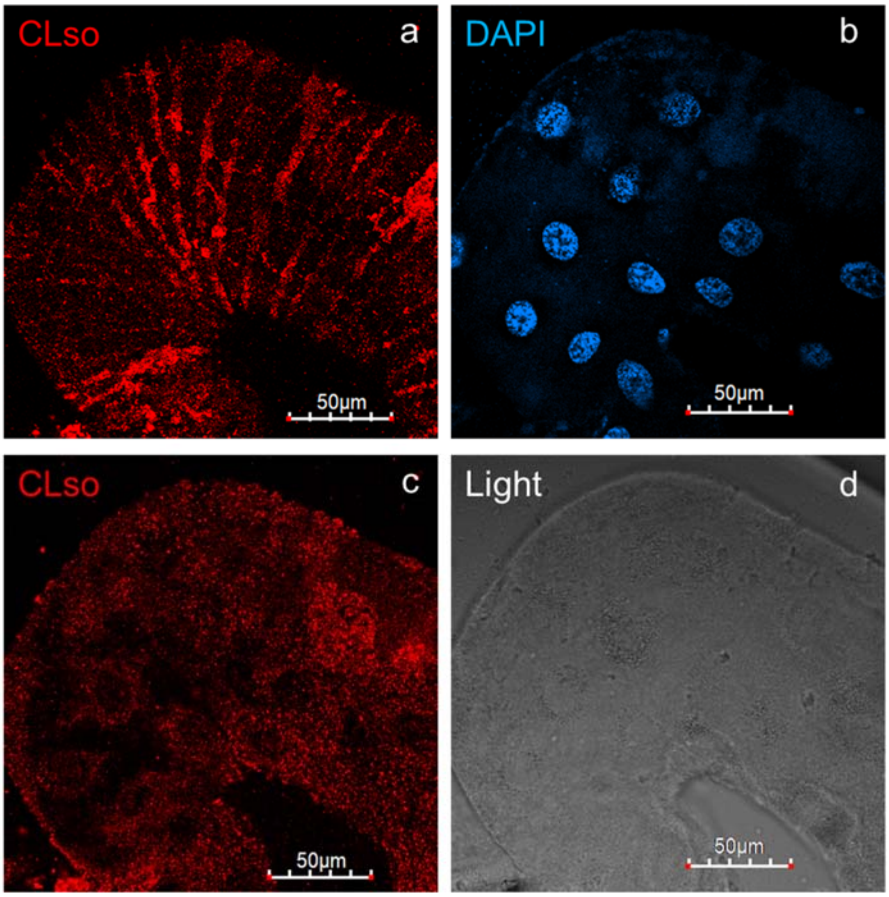


**Figure S4.** Localization of CLso at different focal planes as visualized in the midguts. CLso localizes as stripes at the surface as well as around the ER of the midgut cells.

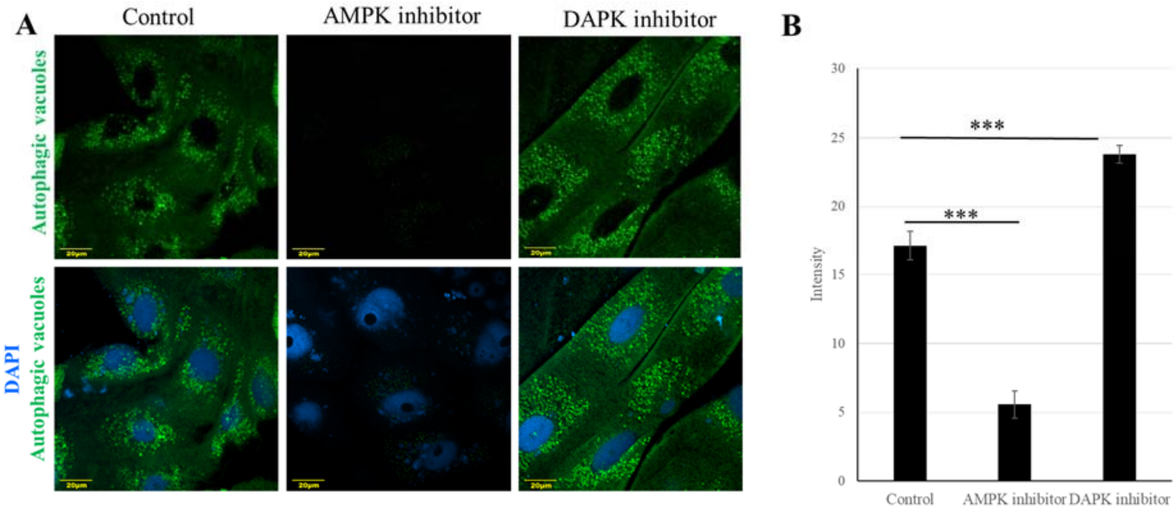


**Figure S5.** Staining of autophagic vacuoles with monodansylcadaverine (MDC) stain after AMPK and DAPK inhibition. A. Confocal images of AMPK inhibition showing reduction in autophagic vacuole formation, whereas there is no difference with DAPK inhibition. B. ImageJ quantification of the MDC stain signals. *** P< 0.0001
